# Helmet noninvasive ventilation for COVID-19 patients (Helmet-COVID): statistical analysis plan for a randomized controlled trial

**DOI:** 10.1186/s13063-021-05988-x

**Published:** 2022-02-02

**Authors:** Yaseen Arabi, Sara Aldekhyl, Saad Al Qahtani, Hasan M. Al-Dorzi, Sheryl Ann Abdukahil, Jesna Jose, Mohammad Khulaif Al Harbi, Husain Al Haji, Mohammed Al Mutairi, Omar Al Zumai, Eman Al Qasim, Wedyan Al Wehaibi, Mohammed Alshahrani, Talal Albrahim, Ahmed Mady, Ali Al Bshabshe, Zohair Al Aseri, Zainab Al Duhailib, Ayman Kharaba, Rakan Alqahtani, Haifa Algethamy, Omar Alfaris, Omar Alnafel, Abdulrahman A. Al-Fares, Haytham Tlayjeh

**Affiliations:** 1 Intensive Care Department, Ministry of National Guard Health Affairs, King Abdullah International Medical Research Center, King Saud Bin Abdulaziz University for Health Sciences, Riyadh, Saudi Arabia; 2 College of Medicine, King Saud Bin Abdulaziz University for Health Sciences, King Abdullah International Medical Research Center, Ministry of National Guard Health Affairs, Riyadh, Saudi Arabia; 3 Bioinformatics and Biostatistics Department, King Abdullah International Medical Research Center, King Saud Bin Abdulaziz University for Health Sciences, Ministry of National Guard Health Affairs, Riyadh, Saudi Arabia; 4 Department of Anesthesia, Ministry of National Guard Health Affairs, King Abdullah International Medical Research Center, King Saud Bin Abdulaziz University for Health Sciences, Riyadh, Saudi Arabia; 5 Respiratory Services Department, Ministry of National Guard Health Affairs, King Abdullah International Medical Research Center, King Saud Bin Abdulaziz University for Health Sciences, Riyadh, Saudi Arabia; 6 Research Office, King Abdullah International Medical Research Center, King Saud Bin Abdulaziz University for Health Sciences, Ministry of National Guard Health Affairs, Riyadh, Saudi Arabia; 7 Department of Emergency and Critical Care, King Fahad Hospital of the University, Imam Abdulrahman Bin Faisal University, Al Khobar, Kingdom of Saudi Arabia; 8 Department of Critical Care, King Fahad Hospital of the University, Imam Abdulrahman Bin Faisal University, Al Khobar, Kingdom of Saudi Arabia; 9 Intensive Care Department, King Saud Medical City, Riyadh, Saudi Arabia; 10 College of Medicine, Tanta University, Tanta, Egypt; 11 Department of Critical Care Medicine, King Khalid University, Aseer Central Hospital, Abha, Kingdom of Saudi Arabia; 12 Emergency and Intensive Care Departments, College of Medicine, King Saud University, Riyadh, Saudi Arabia; 13 Adult Critical Care Department, King Faisal Specialist Hospital & Research Center, Riyadh, Saudi Arabia; 14 Pulmonary & Critical Care Departments, King Fahad Hospital Madinah Critical Care Units, Madinah, Saudi Arabia; 15 Department of Critical Care, College of Medicine, King Saud University, Riyadh, Saudi Arabia; 16 Department of Anesthesia and Critical Care, King Abdulaziz University, King Abdulaziz University Hospital, Jeddah, Saudi Arabia; 17 Respiratory Services Department, Ministry of National Guard Health Affairs, King Abdullah International Medical Research Center, King Saud Bin Abdulaziz University for Health Sciences, Riyadh, Saudi Arabia; 18 Internal Medicine and Intensive Care Department, King Salman Specialist Hospital, Hail, Saudi Arabia; 19 Department of Anesthesia, Critical Care Medicine and Pain Medicine, Al-Amiri Hospital, Ministry of Health, Kuwait, Kuwait

**Keywords:** Noninvasive ventilation, Helmet noninvasive ventilation, COVID-19, Statistical analysis plan

## Abstract

**Background:**

Noninvasive respiratory support is frequently needed for patients with acute hypoxemic respiratory failure due to coronavirus disease 19 (COVID-19). Helmet noninvasive ventilation has multiple advantages over other oxygen support modalities but data about effectiveness are limited.

**Methods:**

In this multicenter randomized trial of helmet noninvasive ventilation for COVID-19 patients, 320 adult ICU patients (aged ≥14 years or as per local standards) with suspected or confirmed COVID-19 and acute hypoxemic respiratory failure (ratio of arterial oxygen partial pressure to fraction of inspired oxygen < 200 despite supplemental oxygen with a partial/non-rebreathing mask at a flow rate of 10 L/min or higher) will be randomized to helmet noninvasive ventilation with usual care or usual care alone, which may include mask noninvasive ventilation, high-flow nasal oxygen, or standard oxygen therapy. The primary outcome is death from any cause within 28 days after randomization. The trial has 80% power to detect a 15% absolute risk reduction in 28-day mortality from 40 to 25%. The primary outcome will be compared between the helmet and usual care group in the intention-to-treat using the chi-square test. Results will be reported as relative risk  and 95% confidence interval. The first patient was enrolled on February 8, 2021. As of August 1, 2021, 252 patients have been enrolled from 7 centers in Saudi Arabia and Kuwait.

**Discussion:**

We developed a detailed statistical analysis plan to guide the analysis of the Helmet-COVID trial, which is expected to conclude enrollment in November 2021.

**Trial registration:**

ClinicalTrials.govNCT04477668. Registered on July 20, 2020

**Supplementary Information:**

The online version contains supplementary material available at 10.1186/s13063-021-05988-x.

## Background

Acute hypoxemic respiratory failure is a common feature of severe coronavirus disease 19 (COVID-19) [1] and frequently requires respiratory support. As invasive mechanical ventilation carries high morbidity and mortality, other respiratory modalities, such as high-flow nasal oxygen (HFNO) and noninvasive ventilation (NIV) delivered via face mask or helmet, have been suggested and increasingly practiced. Helmet NIV has multiple advantages over other modalities that may include more effective seal, less transmission of the virus, more effective delivery of positive end-expiratory pressure (PEEP), and greater tolerance [2, 3]. Helmet NIV has been investigated as a treatment in adult patients with acute hypoxemic respiratory failure [4–8]. A network meta-analysis of 25 studies that included 3804 patients with acute hypoxemic respiratory failure for reasons other than COVID-19 found significantly lower risks of intubation (risk ratio, 0.26; 95% credible interval, 0.14–0.46) and mortality (risk ratio, 0.40; 95% credible interval, 0.24–0.63) with helmet NIV compared with standard oxygen therapy [9]. Recently, a randomized controlled trial (RCT) compared the early application of 48 h of helmet NIV to high-flow nasal oxygen (HFNO) in 109 patients with moderate to severe hypoxemia (ratio of partial pressure of arterial oxygen to fraction of inspired oxygen (PaO_2_:FiO_2_) ratio ≤ 200) and showed no difference in the number of days free of respiratory support at 28 days (primary outcome) with a significantly lower incidence of intubation and a higher number of invasive mechanical ventilation-free days at 28 days in the helmet NIV group [10].

The helmet noninvasive ventilation for COVID-19 patients (Helmet-COVID) trial is a concealed, unblinded multicenter RCT that evaluates the effect of helmet NIV plus usual care compared to usual care alone on 28-day mortality in patients with acute hypoxic respiratory failure due to COVID-19. The full trial protocol has been published previously [11].

In this manuscript, we describe the statistical analysis plan (SAP) of the Helmet-COVID trial. The report describes the procedures for the primary and secondary analyses. All analyses were prospectively defined as the SAP was finalized during trial implementation. The SAP was written by the principal investigator and members of the Steering Committee, who will remain blinded to the study results until all patients have been recruited and the database has been locked. Participant recruitment is expected to be completed in November 2021. The final study report will follow the CONSORT (Consolidated Standards of Reporting Trials) 2010 guidelines for reporting RCTs [12, 13].

## Methods

### Study design

The Helmet-COVID trial will enroll 320 critically ill patients in Saudi Arabia and Kuwait. The study has been approved by the Institutional Review Boards of the participating sites. The trial is registered in ClinicalTrials.gov (NCT04477668). The study is sponsored and funded by King Abdullah International Medical Research Center (protocol number RC20/306/R), Riyadh, Saudi Arabia. The sponsor has no role in the study design, management, or analysis.

### Study population

All adult (adult ICU cut-off age) patients admitted to the ICU with suspected or confirmed COVID-19 by reverse transcription-polymerase chain reaction (RT-PCR) will be screened for eligibility. Inclusion criteria include acute hypoxemic respiratory failure (PaO_2_:FiO_2_ ratio < 200 despite supplemental oxygen with a partial/non-rebreathing mask at a flow rate of 10 L/min or above) with intact airway protective gag reflex and ability to follow instructions. Exclusion criteria include imminent intubation and the requirement for more than one vasopressor. A full list of inclusion and exclusion criteria is described in the published protocol [11]. Enrolled patients will be randomized using a concealed online system to helmet NIV with usual care or usual care alone at a 1:1 ratio. Usual care may include mask NIV, HFNO, or standard oxygen therapy. Randomization will be stratified by site. Helmet NIV will be delivered in pressure support mode, with initial settings of pressure support of 8–10 cm H_2_O, PEEP of 10 cm H_2_O with FiO_2_ of 100%, targeting flow rate of ≥ 50 L/min with an inspiratory rise time of 50 ms, and end flow/cycling off of 50% of maximal inspiratory flow. The settings can be adjusted according to the study protocol. The management of respiratory support in the usual care group including mask NIV is at the discretion of the treating team.

**Fig. 1:**
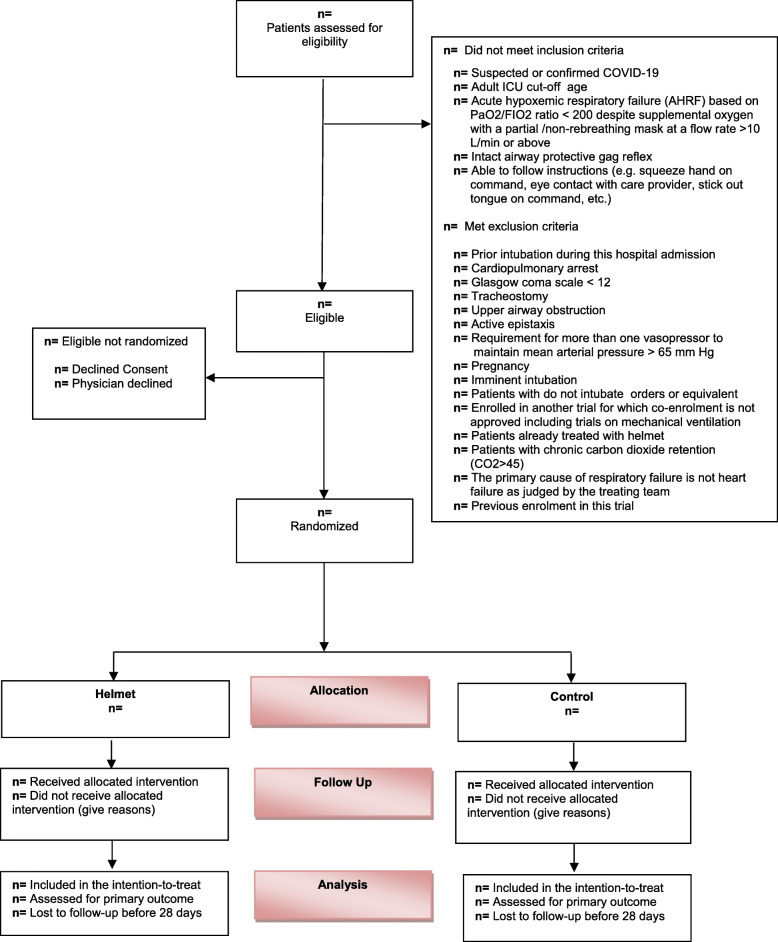
CONSORT flow diagram

A flow diagram will be constructed according to the CONSORT guidelines (Fig. 1). We will report the number of patients who were screened, met inclusion or exclusion criteria, and were eligible but not enrolled with reasons for non-enrollment. We will report the number of patients who were randomized to each group, received the allocated intervention, withdrawn/lost-to-follow-up with reasons, and included in the final analysis.

*The intention-to-treat population* consists of all randomized patients and will be used for the primary analysis. All randomized patients will be included regardless of whether they receive or do not receive the allocated intervention. All patients randomized with suspected COVID-19 will remain in the study, even if they tested negative for COVID-19 after enrollment. Post-enrollment exclusion from the intention-to-treat analysis will be restricted to the withdrawal of consent to use trial data by the patient or surrogate decision-maker (SDM) or wrong randomization (for example, randomization of an ineligible patient in error). However, if the patient or SDM withdraws consent for trial participation but permits collection and use of data, the patient will be included in the intention-to-treat analysis. We plan to enroll additional patients to compensate for patients who are excluded post-randomization, so the sample size of 320 patients in the intention-to-treat cohort is reached.

*The per-protocol population* consists of all randomized patients who receive respiratory support as per the allocated group (helmet NIV in the helmet NIV group, and no helmet NIV in the usual care group). Patients will be considered to have received helmet NIV if the device was applied for 1 h or more.

### Data

#### Baseline characteristics

We will present baseline characteristics in patients randomized to the helmet NIV and usual care groups in the intention-to-treat cohort (Supplement Table S1). We will report in the two groups patients’ age, sex, height, weight, body mass index, location before ICU admission (emergency room, hospital ward, other hospitals (ICU or ward), others), Acute Physiology and Chronic Health Evaluation (APACHE) II, Sequential Organ Failure Assessment (SOFA) score, comorbidities (any chronic comorbidity, chronic cardiac, pulmonary disease, renal, liver, and neurological diseases, diabetes, any malignancy including leukemia or lymphoma and metastatic solid tumor, AIDS/HIV, rheumatologic diseases, others). Because we are enrolling patients with confirmed or suspected COVID-19, we will report whether the patient is eventually confirmed to have COVID-19. We will report physiologic parameters before randomization (PaO_2_:FiO_2_ ratio, partial pressure of carbon dioxide (PaCO_2_), and pH) and the number of quadrants with infiltrates on the chest radiograph. We will report respiratory support at baseline (HFNO, mask NIV, standard oxygen therapy). We will document respiratory rate and whether the patient is treated with awake prone positioning. We will document the number of days from the onset of symptoms to the emergency room and ICU admission and the number of days from ICU admission to randomization. We will report non-respiratory organ support, including vasopressor therapy and renal replacement therapy.

#### Intervention data

For each 24 h in the first 96 h, we will report in each group the details regarding helmet NIV (number of hours used, highest pressure support level, and PEEP). Throughout the first 28 days, we will document the number of days with helmet treatment (> 1 h) and the total hours of helmet NIV. Non-tolerance to helmet NIV is defined as the need to remove the helmet because of patient preference or evidence of clinical deterioration. We will document other reasons for discontinuation of the helmet NIV (clinical improvement, the need for intubation, helmet removal due to change in goals of care or death while on helmet). We will document respiratory support after discontinuation of helmet NIV (mask NIV, HFNO, other oxygen devices, intubation). Violations to the study protocol will be documented including the use of NIV helmet in the usual care group and lack of attempt to use helmet NIV in the intervention group (Supplement Table S2).

#### Co-interventions

In both groups, we will document the use of other respiratory support modalities during the first 4 days (mask NIV with highest pressure support and PEEP, HFNO with flow rate, other oxygen devices, awake prone position), arterial blood gases, and fluid intake and output (Supplement Table S2).

#### Physiologic variables during the intervention

For the 28 days, we will document modalities of respiratory support, the use of sedation while not intubated (dexmedetomidine is permitted in the protocol), renal replacement therapy, and vasopressors/inotrope therapy. We will document the use of COVID-19 treatments, including corticosteroids, IL-6 receptor antagonists, and antiviral therapy. We will document serial arterial oxygen saturation (SaO_2_)/FiO_2_ ratio, fluid balance, and serial SOFA scores (Supplement Table S2).

#### Primary outcome

The primary outcome is all-cause 28-day mortality. The primary outcome tests the primary hypothesis that helmet NIV reduces 28-day mortality (Supplement Table S3).

#### Secondary outcomes

A detailed list of secondary outcomes with definitions has already been published and is outlined in Supplement Table S4. These secondary outcomes can be grouped as follows:

1. Mortality outcomes
  - ICU mortality
  - Hospital mortality
2. Endotracheal intubation. We will document time to intubation, reasons for intubation as determined by the treating team (neurologic deterioration that is not attributed to sedation, persistent or worsening respiratory failure of NIV such as SaO2 < 88%, respiratory rate > 36/min, PaO_2_:FiO_2_ ratio < 100 or persistent requirement of FiO_2_ ≥ 70%, intolerance of mask or helmet NIV, airway bleeding, copious respiratory secretions, respiratory acidosis with pH < 7.25, hemodynamic instability, or significant radiological worsening). We will document mechanical ventilation parameters in the first 24 h after intubation (peak airway pressure, plateau pressure, PEEP in cm H_2_O), FiO_2_, tidal volume, and respiratory rate. We will also document oxygen rescue therapies during invasive mechanical ventilation (neuromuscular blocker infusion, recruitment maneuvers, inhaled nitric oxide, prone positioning, extracorporeal membrane oxygenation (ECMO)). We will report the percentage of patients who underwent tracheostomy.
3. Continuous outcomes
  - ICU-free days at day 28
  - Hospital length of stay (LOS)
  - Invasive mechanical ventilation-free days at day 28
  - Renal replacement therapy-free days at day 28
  - Vasopressor-free days at day 28
4. Safety outcomes
  - Skin injury at the nose, face, neck, and axillae, with the highest stage during the intervention period. We will use the stages as per the National Pressure Ulcer Advisory Panel [14]: stage I: non-blanchable erythema, stage II: partial thickness, stage III: full-thickness skin loss, and stage IV: full-thickness tissue loss
  - Barotrauma, including pneumothorax, mediastinal air or subcutaneous emphysema
  - Cardiovascular events
  - Device complications (such as helmet deflation or malfunction)
  - Serious adverse events (SAEs)
5. Follow-up study: There will be a follow-up of enrolled patients at day 180 about vital status, functional status (EuroQoL (EQ)-5D-5L), by an unblinded assessor, which is planned to be reported separately. For patients who have been discharged from the hospital before day 180, follow-up will be conducted by telephone.

We will also report protocol violations (Supplement Table S6).

### Statistical analysis

Details of sample size calculation have already been published in the study protocol [11]. The sample size of 320 provides a power of 80% to detect a 15% absolute risk reduction in 28-day mortality from 40 to 25%. Categorical variables will be reported as numbers and frequencies and will be compared between the study groups using the chi-square test or Fisher’s exact test. Continuous variables will be reported as means and standard deviations or medians and the first and third quartiles (Q1–Q3) and will be compared between the study groups using the Student’s *t*-test or the Wilcoxon–Mann–Whitney test, as judged appropriate by normality testing using Shapiro–Wilk test. For serial measurements (such as SaO_2_:FiO2 ratio, fluid balance, and serial SOFA), we will test the change over time and the difference between the two study groups over time using generalized linear mixed effect models by considering the link function of logit to incorporate the binary nature of the response variable and link function of log for score outcomes, with no imputation for missing values. To adjust for multiple testing for secondary outcomes and subgroup analyses, we will use the false discovery rate (FDR) as described by Benjamini and Hochberg [15]. We will report associations as relative risk (RR) or hazard ratio (HR) with 95% confidence intervals (CI) or beta coefficient and 95% CI as appropriate. We will compare dyspnea scores and device discomfort scores using generalized linear mixed models, incorporating fixed effects of treatment, time, and the treatment by time interaction and random effects of patient and center. Tests will be two-sided and at the 5% significance level. All statistical analyses will be conducted using the SAS software version 9.4 (SAS Institute, Cary, NC, USA). The statistical analysis remains blinded to the research team until completion of primary outcome data on the study population and will be performed by the study biostatistician. A summary of the analysis plan is provided in Table 1.

**Table 1:** Summary of the analysis plan

Variables	Intention-to-treat cohort	Per-protocol cohort
Baseline characteristics	No statistical comparisons	None
Intervention and co-interventions	Chi-square, Fisher’s exact test, Wilcoxon–Mann–Whitney test, *t*-test as applicable	None
Primary outcome	1. Primary analysis: chi-square or Fisher’s exact test. Report relative risk. 2. Secondary analyses: unadjusted Cox proportional analysis, KM curves, adjusted logistic regression	1. Primary analysis: chi-square or Fisher’s exact test. Report relative risk. 2. Secondary analyses: unadjusted Cox proportional analysis, KM curves, adjusted logistic regression
Secondary outcomes-categorical	Chi-square or Fisher’s exact test. Report relative risk	Chi-square or Fisher’s exact test. Report relative risk
Secondary outcomes-continuous	Generalized linear mixed models. Report beta estimate	Generalized linear mixed models. Report beta estimate
Safety outcomes and other variables	Chi-square or Fisher’s exact test. Report relative risk. For serial measurements, generalized linear mixed effect models	None
Subgroup analyses	Chi-square or Fisher’s exact test. Report relative risk. Tests of interaction	None
180-day follow-up	Chi-square or Fisher’s exact test for 180-day mortality. Report relative risk Generalized linear mixed models for EQ-5D-5L ad VAS. Report beta estimate	Chi-square or Fisher’s exact test for 180-day mortality. Report relative risk Generalized linear mixed models for EQ-5D-5L ad VAS. Report beta estimate

#### Analysis of primary outcome

The primary outcome will be compared in the intention-to-treat using the chi-square test. Results will be reported as RR with 95% CI. We will use an unadjusted Cox proportional hazard model as a secondary analysis tool, and the results will be reported as hazard ratio (HR) and 95% CI. We will use Kaplan–Meier survival function estimates to assess proportional hazards for categorical covariates. Moreover, we will be using the supremum test of the null hypothesis that the observed pattern of martingale residuals is not different from the expected pattern [16]. A very small *p*-value (≤ 0.05) suggests a violation of proportional hazards. Kaplan–Meier curves will be generated for the two study groups and a log-rank test will be used to compare distributions. Although imbalances in baseline characteristics are unlikely with the large sample size, we will conduct an adjusted logistic regression model to adjust for the following factors (defined a priori): enrolment center, respiratory support at baseline (mask NIV support versus others), PaO_2_:FiO_2_ ratio, body mass index > 30 kg/m^2^, age, APACHE II score, and time (time of enrolling the first half of the cohort versus the second half); the latter will be included to account for the changes in outcomes of COVID-19 patients during the pandemic.

#### Analysis of secondary outcomes

Secondary outcomes will be compared in the intention-to-treat cohort only. Categorical outcomes will be compared in the intention-to-treat using a chi-square test. Results will be reported as RR with 95% CI. Continuous outcomes will be compared using generalized linear mixed models. Results will be reported as beta estimates with 95% CI.

#### Subgroup analyses

The primary outcome will be compared in the intention-to-treat cohort only, in the following a priori defined subgroups using a chi-square test.

1. PaO_2_:FiO_2_ ratio 101–200 and PaO_2_:FiO_2_ ratio < 100
2. Obese patients (body mass index > 30 kg/m^2^) and patients with a body mass index of ≤ 30 kg/m^2^
3. Patients aged > 65 years and ≤ 65 years
4. APACHE II score higher or lower than the median of enrolled patients
5. Patients who were at the time of enrollment on mask NIV versus other types of respiratory support

Results will be reported using RR and 95% CI. We will report the results of the test of interactions for these subgroups (Supplement Table S5).

#### Sensitivity analyses

We will compare the primary outcome between the helmet NIV and usual care groups in the per-protocol cohorts (effectiveness analysis). If patients with suspected COVID-19 who tested negative constituted more than 5% of the study population, we will carry a sensitivity analysis excluding these patients.

#### 180-day follow-up study

In a follow-up report, we will compare 180-day mortality and the EuroQol (EQ-5D-5L) [17] at 6-month scores after randomization between the two study groups. The EQ-5D-5L scores will be converted into a single index value that generates a measure of utility ranging from −0.111 to 1.000 (1.000 indicates full health) using an online tool [18, 19]. We will use generalized linear mixed models to compare EQ-5D-5L index values and VAS scores between the two groups by incorporating treatment, baseline values, and random patient and center effects in the model.

#### Interim analyses

The interim test statistics will be conducted for the primary outcome. Two formal interim analyses are planned when 33% and 67% of the sample size are reached. The trial may be stopped for safety (*p* < 0.01) or effectiveness (*p* < 0.001) both evaluating the primary outcome (28-day mortality) but there will be no plans to terminate the trial for futility. We will account for alpha spending by the O’Brien-Fleming method and the final *p*-value will be considered at 0.048 [20].

## Discussion

Several studies have investigated helmet NIV as a treatment for acute hypoxic respiratory failure [4–8]. A systematic review of RCTs and observational studies found that helmet NIV was associated with lower hospital mortality (odds ratio, 0.43; 95% *CI*, 0.26–0.69), intubation rate (odds ratio, 0.32; 95% *CI*, 0.21–0.47), and complications (odds ratio, 0.6; 95% *CI*, 0.4–0.92) compared with controls [21]. A meta-analysis of four RCTs (377 patients) showed that helmet NIV significantly increased the PaO_2_:FiO_2_ (+ 73.4; 95% *CI*, 43.9–102.9) and decreased the arterial CO_2_ levels, intubation rate (RR, 0.21; 95% *CI*, 0.11–0.40), and in-hospital mortality rate (RR, 0.22; 95% *CI*, 0.09–0.50) compared to standard oxygen therapy [22]. A network meta-analysis of 25 studies that included 3804 patients with acute hypoxemic respiratory failure for reasons other than COVID-19 found significantly lower intubation risks with helmet NIV compared with standard oxygen therapy [9]. The advantages of helmet NIV over other oxygen support modalities are thought to be more prominent in patients with COVID-19. This led to the design and conduct of multiple RCTs [10, 23]. Recently, one trial (*n* = 110) showed no difference in the number of days free of respiratory support at 28 days (primary outcome) between helmet NIV and HFNO [10].

The trial is unblinded given the nature of the intervention. The use of an objective, all-cause mortality as the primary outcome mitigates concerns regarding outcome assessment. The lack of blinding for the long-term quality of life outcomes is a limitation.

As the efficacy of helmet NIV to improve outcomes in severe acute hypoxemic respiratory failure due to COVID-19 pneumonia has not been established, the aim of the Helmet-COVID trial is to compare the effectiveness of helmet NIV compared to usual care on 28-day mortality in patients with acute hypoxic respiratory failure from COVID-19. The results of the trial and the possibility of contributing to individual patient meta-analysis would likely help address this important question.

## Trial status

The first patient was enrolled on February 8, 2021, and enrollment is expected to be concluded in November 2021.

## Conclusion

The Helmet-COVID trial evaluates whether helmet NIV improves the outcomes of critically ill patients with acute hypoxemic respiratory failure due to COVID-19. It is expected to provide evidence that will inform practice regarding the use of helmet NIV for respiratory support in these patients and contribute to future clinical practice guidelines.

## Supplementary Information

**Additional file 1.** Supplementary tables and figures

## Data Availability

The dataset will be available from the corresponding author upon reasonable request in accordance with the regulations of King Abdullah International Medical Research Center, Riyadh, Saudi Arabia.

## Abbreviations

CI: Confidence interval
HR: Hazard ratio
ICU: Intensive care unit
IQR: Interquartile range
LOS: Length of stay
RT-PCR: Reverse transcription–polymerase chain reaction
RCT: Randomized controlled trial
RR: Relative risk
RRR: Relative risk reduction
SAP: Statistical analysis plan
