# Supplementary material for: Helmet noninvasive ventilation for COVID-19 patients (Helmet-COVID): statistical analysis plan for a randomized controlled trial
Source: Trials. 2022 Feb 2;23:105. doi: 10.1186/s13063-021-05988-x (PMC8808278; doi:10.1186/s13063-021-05988-x)
Supplement: Supplementary file 1 — Additional file 1. Supplementary tables and figures [file 13063_2021_5988_MOESM1_ESM.docx]

**Supplementary file to helmet noninvasive ventilation for COVID-19 patients (Helmet-COVID): statistical analysis plan for a randomized controlled trial**

Appendix A Supplementary Tables …………………………………………………………………...2

Appendix B Supplementary Figures …………………………………………………………………. 13

Appendix C Data Management and Standard Operating Procedures …………………………… 14

**Appendix A**

**Table S1:** Baseline characteristics – ITT population.

| **Characteristic** | **Helmet NIV (N=XXX)** | **Usual care (N=XXX)** |
| --- | --- | --- |
| Age (Years) - Mean (SD) | xx (xx.x) | xx (xx.x) |
| Male sex - n (%) | xxx/xxx (xx.x) | xxx/xxx (xx.x) |
| Height (cm) - Median (Q1,Q3) | xx (xx.x) | xx (xx.x) |
| Weight (kg) - Median (Q1,Q3) | xx (xx, xx) | xx (xx, xx) |
| BMI (kg/m^2^) - Median (Q1,Q3) | xx (xx, xx) | xx (xx, xx) |
|  |  |  |
| Location prior to ICU admission - n (%) |  |  |
| Emergency room | xxx (xx.x) | xxx (xx.x) |
| Hospital ward | xxx (xx.x) | xxx (xx.x) |
| Other hospital (ICU or ward) | xxx (xx.x) | xxx (xx.x) |
| Other | xxx (xx.x) | xxx (xx.x) |
|  |  |  |
| APACHE II- Median (Q1,Q3) | xx (xx, xx) | xx (xx, xx) |
| SOFA score- Median (Q1,Q3) | xx (xx, xx) | xx (xx, xx) |
|  |  |  |
| Comorbidities - n (%) |  |  |
| Any chronic comorbidity | xxx (xx.x) | xxx (xx.x) |
| Chronic cardiac disease | xxx (xx.x) | xxx (xx.x) |
| Chronic pulmonary disease | xxx (xx.x) | xxx (xx.x) |
| Chronic renal disease | xxx (xx.x) | xxx (xx.x) |
| Mild, moderate or severe liver disease | xxx (xx.x) | xxx (xx.x) |
| Chronic neurological disease, hemiplegia or paraplegia, or dementia | xxx (xx.x) | xxx (xx.x) |
| Diabetes | xxx (xx.x) | xxx (xx.x) |
| Any malignancy including leukemia or lymphoma and metastatic solid tumor | xxx (xx.x) | xxx (xx.x) |
| AIDS/HIV | xxx (xx.x) | xxx (xx.x) |
| Rheumatologic diseases | xxx (xx.x) | xxx (xx.x) |
| Others | xxx (xx.x) | xxx (xx.x) |
|  |  |  |
| Confirmed SARS-CoV-2 infection* n/N (%) | xxx/xxx (xx.x) | xxx/xxx (xx.x) |
|  |  |  |
| Physiologic parameters prior to randomization–Median (Q1,Q3) |  |  |
| PaO_2_ (mmHg) | xxx (xx.x) | xxx (xx.x) |
| FiO_2_ | xxx (xx.x) | xxx (xx.x) |
| PaO_2_:FiO_2_ ratio | xxx (xx.x) | xxx (xx.x) |
| PCO_2_ (mmHg) | xxx (xx.x) | xxx (xx.x) |
| HCO_3_ | xxx (xx.x) | xxx (xx.x) |
| pH | xxx (xx.x) | xxx (xx.x) |
| SaO2:FiO2 | xxx (xx.x) | xxx (xx.x) |
| Number of quadrants with infiltrates on chest radiograph | xxx (xx.x) | xxx (xx.x) |
|  |  |  |
| Respiratory support at baseline- n (%) |  |  |
| High flow nasal cannula | xxx/xxx (xx.x) | xxx/xxx (xx.x) |
| Noninvasive ventilation | xxx/xxx (xx.x) | xxx/xxx (xx.x) |
| Others | xxx/xxx (xx.x) | xxx/xxx (xx.x) |
|  |  |  |
| Respiratory rate (breaths/minute) –Median (Q1,Q3) | xxx (xx.x) | xxx (xx.x) |
| Awake proning- n (%) | xxx/xxx (xx.x) | xxx/xxx (xx.x) |
|  |  |  |
| Days from onset of symptoms to the emergency room— Median (Q1, Q3) | xxxx (xx.x) | xxxx (xx.x) |
| Days from onset of symptoms to ICU admission — Median (Q1, Q3) | xxxx (xx.x) | xxxx (xx.x) |
| Number of days from ICU admission to randomization — Median (Q1, Q3) | xx (xx, xx) | xx (xx, xx) |
|  |  |  |
| Organ support - n (%) |  |  |
| Vasopressors | xxx/xxx (xx.x) | xxx/xxx (xx.x) |
| Renal replacement therapy | xxx/xxx (xx.x) | xxx/xxx (xx.x) |

Denominator of the percentage is the total number of subjects in each group in the ITT population.

ITT: Intention-to-treat, APACHE: Acute physiology and chronic health evaluation II, Intensive care unit, FiO_2_: denotes the fraction of inspired oxygen, PaO_2_: partial pressure of oxygen in arterial blood, PaCO_2_: partial pressure of carbon dioxide, SOFA: Sequential organ failure assessment.

*Infection is confirmed by a respiratory tract polymerase-chain-reaction test.

**Table S2:** Summary of interventions and co-interventions in the intention-to-treat population.

| **Variable** | **Helmet NIV (N=XXX)** | **Usual care (N=XXX)** |
| --- | --- | --- |
| Use of helmet |  |  |
| Number of patients receiving helmet during the study period – no. (%) | xxx (xx.x) | xxx (xx.x) |
| Helmet use | xx (xx, xx) | xx (xx, xx) |
| Day 1 number of hours used - Mean (SD) | xx (xx.x) | xx (xx.x) |
| Highest pressure support level - Median (Q1, Q3) | xx (xx, xx) | xx (xx, xx) |
| Highest PEEP - Median (Q1, Q3) | xx (xx, xx) | xx (xx, xx) |
| Day 2 number of hours used - Mean (SD) | xx (xx.x) | xx (xx.x) |
| Highest pressure support level - Median (Q1, Q3) | xx (xx, xx) | xx (xx, xx) |
| Highest PEEP - Median (Q1, Q3) | xx (xx, xx) | xx (xx, xx) |
| Day 3 number of hours used - Mean (SD) | xx (xx.x) | xx (xx.x) |
| Highest pressure support level - Median (Q1, Q3) | xx (xx, xx) | xx (xx, xx) |
| Highest PEEP - Median (Q1, Q3) | xx (xx, xx) | xx (xx, xx) |
| Day 4 number of hours used - Mean (SD) | xx (xx.x) | xx (xx.x) |
| Highest pressure support level - Median (Q1, Q3) | xx (xx, xx) | xx (xx, xx) |
| Highest PEEP - Median (Q1, Q3) | xx (xx, xx) | xx (xx, xx) |
| Number of days received helmet (>1 hour) - Mean (SD) | xx (xx.x) | xx (xx.x) |
| Total hours of helmet - Mean (SD) | xx (xx.x) | xx (xx.x) |
|  |  |  |
| Reasons for discontinuation of helmet– no. (%) | xxx (xx.x) | xxx (xx.x) |
| Clinical improvement | xxx (xx.x) | xxx (xx.x) |
| Patient required intubation | xxx (xx.x) | xxx (xx.x) |
| Intolerance and helmet use <1 hour |  |  |
| Intolerance and helmet use >1 hour | xxx (xx.x) | xxx (xx.x) |
| Helmet removal due to change in goals of care | xxx (xx.x) | xxx (xx.x) |
| Death while helmet was on | xxx (xx.x) | xxx (xx.x) |
|  |  |  |
| Other respiratory support during the first 4 days |  |  |
| Mask NIV– no. (%) | xxx (xx.x) | xxx (xx.x) |
| Highest pressure support level on day 1 - Median (Q1, Q3) | xx (xx, xx) | xx (xx, xx) |
| Highest PEEP on day 1 - Median (Q1, Q3) | xx (xx, xx) | xx (xx, xx) |
| High flow nasal cannula – no. (%) | xxx (xx.x) | xxx (xx.x) |
| Flow rate on day 1 - Median (Q1, Q3) | xx (xx, xx) | xx (xx, xx) |
| Other oxygen devices | xxx (xx.x) | xxx (xx.x) |
| Awake proning – no. (%) | xxx (xx.x) | xxx (xx.x) |
|  |  |  |
| Co-Interventions – no. (%) |  |  |
| Use of sedation (dexmedetomidine) during NIV support | xxx (xx.x) | xxx (xx.x) |
| Renal replacement therapy | xxx (xx.x) | xxx (xx.x) |
| Vasopressors/Inotropes | xxx (xx.x) | xxx (xx.x) |
|  |  |  |

| **Variable** | **Helmet NIV (N=XXX)** | **Usual care (N=XXX)** |
| --- | --- | --- |
| COVID-19 interventions – no. (%) |  |  |
| Hydroxychloroquine | xxx (xx.x) | xxx (xx.x) |
| Chloroquine | xxx (xx.x) | xxx (xx.x) |
| Macrolide | xxx (xx.x) | xxx (xx.x) |
| Lopinavir/Ritonavir | xxx (xx.x) | xxx (xx.x) |
| Favipiravir | xxx (xx.x) | xxx (xx.x) |
| Remdesivir | xxx (xx.x) | xxx (xx.x) |
| Ribavirin | xxx (xx.x) | xxx (xx.x) |
| Intravenous immunoglobulin | xxx (xx.x) | xxx (xx.x) |
| Interferon | xxx (xx.x) | xxx (xx.x) |
| Oseltamivir | xxx (xx.x) | xxx (xx.x) |
| Beta-lactam/Beta-lactamase inhibitor | xxx (xx.x) | xxx (xx.x) |
| Tocilizumab | xxx (xx.x) | xxx (xx.x) |
| Convalescent plasma | xxx (xx.x) | xxx (xx.x) |
| Steroids | xxx (xx.x) | xxx (xx.x) |
| Hydrocortisone | xxx (xx.x) | xxx (xx.x) |
| Dexamethasone | xxx (xx.x) | xxx (xx.x) |
| Prednisolone | xxx (xx.x) | xxx (xx.x) |
| Methyprednisolone | xxx (xx.x) | xxx (xx.x) |
| Other treatments | xxx (xx.x) | xxx (xx.x) |

Calculations are provided for the all patients in each group.

CRRT: continuous renal replacement therapy, FiO_2_: denotes the fraction of inspired oxygen, NIV: non-invasive ventilation, PaO_2_: partial pressure of oxygen in arterial blood, PaCO_2_: partial pressure of carbon dioxide, PEEP: Positive end-expiratory pressure, SOFA: Sequential organ failure assessment

.

**Table S3:** Primary outcome: 28-day mortality.

|  | **Intention-to-treat population** | | | **Per-protocol population** | | |
| --- | --- | --- | --- | --- | --- | --- |
| **Variable** | **Helmet NIV (N=XXX)** | **Usual care (N=XXX)** | **P-value** | **Helmet NIV (N=XXX)** | **Usual care (N=XXX)** | **P-value** |
| 28-day mortality, n (%) | xx/xxx (xx.x) | xx/xxx (xx.x) | x.xxx | xx/xxx (xx.x) | xx/xxx (xx.x) | x.xxx |
| Relative Risk, (95% CI) | xx.x (xx.x, xx.x) | |  | xx.x (xx.x, xx.x) | |  |
| Days to event - Median (Q1,Q3) | xx (xx, xx) | xx (xx, xx) |  | xx (xx, xx) | xx (xx, xx) |  |
| Unadjusted hazard Ratio (95% CI) | x.xx (x.xx, x.xx) | | x.xxx | x.xx (x.xx, x.xx) | | x.xxx |
| Adjusted relative risk, (95% CI) | x.xx (x.xx, x.xx) | | x.xxx | x.xx (x.xx, x.xx) | | x.xxx |

**Table S4:** Secondary and safety outcomes ITT population.

| **Variable** | **Helmet NIV (N=XXX)** | **Usual care (N=XXX)** | **Relative risk/Beta estimate (95% CI)** | **P-value** |
| --- | --- | --- | --- | --- |
| 28-day mortality – n (%) | xxxx (xx.x) | xxxx (xx.x) | x.xx (x.xx , x.xx) | x.xxx |
| ICU mortality – n (%) | xxxx (xx.x) | xxxx (xx.x) | x.xx (x.xx , x.xx) | x.xxx |
| Hospital mortality – n (%) | xxxx (xx.x) | xxxx (xx.x) | x.xx (x.xx , x.xx) | x.xxx |
| 180-day mortality – n (%) | xxxx (xx.x) | xxxx (xx.x) | x.xx (x.xx , x.xx) | x.xxx |
|  |  |  |  |  |
| ICU-free days at day 28 | xx (xx, xx) | xx (xx, xx) | x.xx (x.xx , x.xx) | x.xxx |
| Hospital LOS - Median (Q1,Q3) | xx (xx, xx) | xx (xx, xx) | x.xx (x.xx , x.xx) | x.xxx |
|  |  |  |  |  |
| Mechanical Ventilation free days - Median (Q1,Q3) | xx (xx, xx) | xx (xx, xx) | x.xx (x.xx , x.xx) | x.xxx |
| Renal replacement-free days at day 28 | xx (xx, xx) | xx (xx, xx) | x.xx (x.xx , x.xx) | x.xxx |
| Vasopressor-free days at day 28 | xx (xx, xx) | xx (xx, xx) | x.xx (x.xx , x.xx) | x.xxx |
|  |  |  |  |  |
| Endotracheal intubation – n (%) | xxx (xx.x) | xxx (xx.x) | x.xx (x.xx , x.xx) | x.xxx |
| Time to intubation - Median (Q1,Q3) | xx (xx, xx) | xx (xx, xx) | x.xx (x.xx , x.xx) | x.xxx |
| Reasons for intubation – n (%) |  |  |  |  |
| Neurologic deterioration (not attributed to sedation) | xxx (xx.x) | xxx (xx.x) | x.xx (x.xx , x.xx) | x.xxx |
| Persistent or worsening respiratory failure of NIV | xxx (xx.x) | xxx (xx.x) | x.xx (x.xx , x.xx) | x.xxx |
| Oxygen saturation <88% | xxx (xx.x) | xxx (xx.x) | x.xx (x.xx , x.xx) | x.xxx |
| Respiratory rate >36/min | xxx (xx.x) | xxx (xx.x) | x.xx (x.xx , x.xx) | x.xxx |
| P/F ratio <100 | xxx (xx.x) | xxx (xx.x) | x.xx (x.xx , x.xx) | x.xxx |
| Persistent requirement of FiO2 ≥70% | xxx (xx.x) | xxx (xx.x) | x.xx (x.xx , x.xx) | x.xxx |
| Intolerance of face mask or helmet | xxx (xx.x) | xxx (xx.x) | x.xx (x.xx , x.xx) | x.xxx |
| Airway bleeding | xxx (xx.x) | xxx (xx.x) | x.xx (x.xx , x.xx) | x.xxx |
| Copious respiratory secretions | xxx (xx.x) | xxx (xx.x) | x.xx (x.xx , x.xx) | x.xxx |
| Respiratory acidosis with pH <7.25 | xxx (xx.x) | xxx (xx.x) | x.xx (x.xx , x.xx) | x.xxx |
| Hemodynamic instability | xxx (xx.x) | xxx (xx.x) | x.xx (x.xx , x.xx) | x.xxx |
| Significant radiologic worsening | xxx (xx.x) | xxx (xx.x) | x.xx (x.xx , x.xx) | x.xxx |
|  |  |  |  |  |
| Mechanical ventilation parameters in the first 24 hours of intubation |  |  |  |  |
| Ppeak pressure (cmH_2_O) - Median (Q1,Q3) | xx (xx, xx) | xx (xx, xx) |  | x.xxx |
| Pplateau (if done) - Median (Q1,Q3) | xx (xx, xx) | xx (xx, xx) |  | x.xxx |
| PEEP (cmH_2_O) - Median (Q1,Q3) | xx (xx, xx) | xx (xx, xx) |  | x.xxx |
| FiO_2_ (%)- Median (Q1,Q3) | xx (xx, xx) | xx (xx, xx) |  | x.xxx |
| Tidal volume (ml) - Median (Q1,Q3) | xx (xx, xx) | xx (xx, xx) |  | x.xxx |
| Respiratory rate (breaths/min) - Median (Q1,Q3) | xx (xx, xx) | xx (xx, xx) |  | x.xxx |
|  |  |  |  |  |
| Therapies received during invasive mechanical ventilation – n (%) |  |  |  |  |
| Neuromuscular blocker infusion | xxx (xx.x) | xxx (xx.x) | x.xx (x.xx , x.xx) | x.xxx |
| Recruitment maneuvers | xxx (xx.x) | xxx (xx.x) | x.xx (x.xx , x.xx) | x.xxx |
| Inhaled Nitric oxide | xxx (xx.x) | xxx (xx.x) | x.xx (x.xx , x.xx) | x.xxx |
| Prone positioning | xxx (xx.x) | xxx (xx.x) | x.xx (x.xx , x.xx) | x.xxx |
| ECMO | xxx (xx.x) | xxx (xx.x) | x.xx (x.xx , x.xx) | x.xxx |
|  |  |  |  |  |
| Tracheostomy – n (%) | xxx (xx.x) | xxx (xx.x) | x.xx (x.xx , x.xx) | x.xxx |
|  |  |  |  |  |
| Safety outcomes |  |  |  |  |
| Skin ulceration at nose, face, neck and axillae (highest stage during intervention period) |  |  |  |  |
| Stage I: Non-blanchable erythema | xxx (xx.x) | xxx (xx.x) | x.xx (x.xx , x.xx) | x.xxx |
| Stage II: Partial thickness | xxx (xx.x) | xxx (xx.x) | x.xx (x.xx , x.xx) | x.xxx |
| Stage III: Full thickness skin loss | xxx (xx.x) | xxx (xx.x) | x.xx (x.xx , x.xx) | x.xxx |
| Stage IV: Full thickness tissue loss | xxx (xx.x) | xxx (xx.x) | x.xx (x.xx , x.xx) | x.xxx |
| Barotrauma | xxx (xx.x) | xxx (xx.x) | x.xx (x.xx , x.xx) | x.xxx |
| Pneumothorax | xxx (xx.x) | xxx (xx.x) | x.xx (x.xx , x.xx) | x.xxx |
| Mediastinal air | xxx (xx.x) | xxx (xx.x) | x.xx (x.xx , x.xx) | x.xxx |
| Subcutaenous emphysema | xxx (xx.x) | xxx (xx.x) | x.xx (x.xx , x.xx) | x.xxx |
| Cardiovascular events | xxx (xx.x) | xxx (xx.x) | x.xx (x.xx , x.xx) | x.xxx |
| Device complication (helmet deflation) | xxx (xx.x) | xxx (xx.x) | x.xx (x.xx , x.xx) | x.xxx |
|  |  |  |  |  |
| Serious adverse events (SAEs) | xxx (xx.x) | xxx (xx.x) | x.xx (x.xx , x.xx) | x.xxx |
| SAE 1 | xxx (xx.x) | xxx (xx.x) | x.xx (x.xx , x.xx) | x.xxx |
| SAE 2 | xxx (xx.x) | xxx (xx.x) | x.xx (x.xx , x.xx) | x.xxx |
| SAE 3 | xxx (xx.x) | xxx (xx.x) | x.xx (x.xx , x.xx) | x.xxx |

Denominator of the percentage is the total number of subjects in each group in the ITT and PP population.

Mechanical Ventilation free days Vasopressor free days and ICU free days are calculated based on 28-d observation

LOS: Length of Stay, ECMO: Extracorporeal membrane oxygenation; ICU: Intensive care unit

For continuous variables, the Mann-Whitney U test was used to calculate p value

For categorical variables, the Fishers exact test was used to calculate p value

**Table S5:** Subgroup analyses.

|  | **28-day mortality** | | | | |
| --- | --- | --- | --- | --- | --- |
|  | **Helmet NIV (N=XXX)** | **Usual care (N=XXX)** | **RR (95% CI)** | **P-value** | **P-value for interaction** |
| PaO2:FiO2 <100 | xxx/xxx (xx.x) | xxx/xxx (xx.x) | x.xx (x.xx, x.xx) | x.xxx | x.xxx |
| PaO2:FiO2 100-200 | xxx/xxx (xx.x) | xxx/xxx (xx.x) | x.xx (x.xx, x.xx) | x.xxx |  |
|  |  |  |  |  |  |
| BMI >30 | xxx/xxx (xx.x) | xxx/xxx (xx.x) | x.xx (x.xx, x.xx) | x.xxx | x.xxx |
| BMI ≤30 | xxx/xxx (xx.x) | xxx/xxx (xx.x) | x.xx (x.xx, x.xx) | x.xxx |  |
|  |  |  |  |  |  |
| Age >65 | xxx/xxx (xx.x) | xxx/xxx (xx.x) | x.xx (x.xx, x.xx) | x.xxx | x.xxx |
| Age ≤65 | xxx/xxx (xx.x) | xxx/xxx (xx.x) | x.xx (x.xx, x.xx) | x.xxx |  |
|  |  |  |  |  |  |
| APACHE II >Median | xxx/xxx (xx.x) | xxx/xxx (xx.x) | x.xx (x.xx, x.xx) | x.xxx | x.xxx |
| APACHE II ≤Median | xxx/xxx (xx.x) | xxx/xxx (xx.x) | x.xx (x.xx, x.xx) | x.xxx |  |

ARDS: Acute respiratory distress syndrome, BMI: Body mass index, APACHE: Acute physiology and chronic health evaluation II

**Table S6:** Summary of protocol violations.

| **Characteristics** | **Helmet NIV (N=XXX)** | **Usual care (N=XXX)** |
| --- | --- | --- |
|  |  |  |
| Protocol violations | xx (xx.x) | xx (xx.x) |
| xxxxxxx | xx (xx.x) | xx (xx.x) |
| xxxxxxx | xx (xx.x) | xx (xx.x) |
|  |  |  |
| Reasons for protocol violations |  |  |
| xxxxxxx | xx (xx.x) | xx (xx.x) |
| xxxxxxx | xx (xx.x) | xx (xx.x) |
|  |  |  |
| Any consequences |  |  |
| Yes | xx (xx.x) | xx (xx.x) |
| No | xx (xx.x) | xx (xx.x) |
| Denominator of the percentage is the total number of patients in the treatment group. | | |

**Table S7:** EQ-5D-5L at baseline and day 180 post randomization.

|  | **Baseline** | | | | **180-day** | | |
| --- | --- | --- | --- | --- | --- | --- | --- |
| **Variable** | **Helmet NIV (N=XXX)** | **Usual care (N=XXX)** | **P value** | **Helmet NIV (N=XXX)** | | **Usual care (N=XXX)** | **P value** |
| Mobility, Median (Q1,Q3) | Xx (xx, xx) | Xx (xx, xx) | x.xxx | Xx (xx, xx) | | Xx (xx, xx) | x.xxx |
| Self-care , Median (Q1,Q3) | Xx (xx, xx) | Xx (xx, xx) | x.xxx | Xx (xx, xx) | | Xx (xx, xx) | x.xxx |
| Usual activities, Median (Q1,Q3) | Xx (xx, xx) | Xx (xx, xx) | x.xxx | Xx (xx, xx) | | Xx (xx, xx) | x.xxx |
| Pain discomfort, Median (Q1,Q3) | Xx (xx, xx) | Xx (xx, xx) | x.xxx | Xx (xx, xx) | | Xx (xx, xx) | x.xxx |
| Anxiety depression, Median (Q1,Q3) | Xx (xx, xx) | Xx (xx, xx) | x.xxx | Xx (xx, xx) | | Xx (xx, xx) | x.xxx |
|  |  |  |  |  | |  |  |
| EQ-5D-5L index values, Median (Q1,Q3) | Xx (xx, xx) | Xx (xx, xx) | x.xxx | Xx (xx, xx) | | Xx (xx, xx) | x.xxx |
| Beta Estimate (CI) |  |  |  | x.xx (x.xx, x.xx) | | |  |
|  |  |  |  |  | |  |  |
| VAS (QoL) scores, Median (Q1,Q3) | Xx (xx, xx) | Xx (xx, xx) | x.xxx | Xx (xx, xx) | | Xx (xx, xx) | x.xxx |
|  |  |  |  |  | |  |  |
| Beta Estimate (CI) |  |  |  | x.xx (x.xx, x.xx) | | |  |

**Appendix B**

**Figures**

**Figure 1:** Kaplan Meier curve for mortality

**Figure 2:** Kaplan Meier curve for time to intubation

**Figure 3:** Serial SOFA, Serial SaO2:FiO2, Serial FiO2, Serial fluid balance

**Figure 4:** Visual Analog Scale – Dyspnea, Visual Analog Scale – Device discomfort

**Figure 5:** EQ-5D-5L and VAS at baseline and at Day 180 (follow-up study)

**Appendix C**

**Data management plan**

Data are entered through a password-protected access to an electronic database through an online portal and is stored on a secure server at King Abdullah International Medical Research Center, Riyadh, Saudi Arabia. The database includes multiple logic checks for double entry and range checks for data values. Several procedures to ensure data quality and protocol standardization are undertaken including: 1) training sessions for research coordinators from participating centers prior to study commencement; 2) a detailed study instruction manual which outlines each step of the protocol; 3) startup meetings for all sites, either by a physical visit or via videoconferencing. Patient personal data are de-identified. Each site investigator and coordinator have access to data of patients from their site; the PI and the main coordinator has access to the data from all sites.

**Helmet-NIV Protocol**

**Setup and preparation**

- Use unheated, low-compliance, dual limb breathing circuit.
- Insert a bacterial/viral filter at the expiratory port of the ventilator.
- Carry out the required preoperational check.
- To avoid patient-ventilator asynchrony, explain the application and the procedure of the helmet to the patient.
- Insert the earplugs into patient ears.
- Use the under-arm pads to avoid strap pressure.

**Initial setup**

- Can be used with a ventilator or NIV machines.
- Set up the ventilator on Pressure Support Ventilation (PSV).
- PSV 8-10 cm H2O pressure, positive end-expiratory pressure (PEEP) 10 cm H2O pressure, FiO2 100%.
- Flow rate >50 L/min, inspiratory rise time 50 msec, End Flow/Cycling off 50% of maximal inspiratory flow.

**Titration**

- Increase PEEP by 2-3 cm H2O every 3 minutes to achieve SpO2 ≥ 92% on FiO2 ≤ 60%
- Higher PEEP than what is used on face mask NIV is allowed and tolerated.
- Increase PS by 2-3 cm every 3 minutes to achieve respiratory rate (RR) ≤ 25/min or clear patient comfort.
- The maximal allowed PS + PEEP is 30 cm H_2_O.
- Helmet should be applied continuously for at least 48 hours.
- Titrate FiO2 to ≤60% as soon as possible.

**Sedation**

- Dexmedetomidine may be used to improve compliance with the helmet. However, other intravenous sedatives such as benzodiazepines or intravenous narcotics should not be used.

**Weaning when clinical condition allows**

- Titrate PS by 2 cm H2O every 3 hours if RR ≤ 25/min.
- Titrate PEEP by 2 cm H2O every 3 hours if SpO2 >92% on <60% FiO2.
- If RR ≤25/min on PSV ≤8 cm and SpO2 >92% on FiO2 ≤50% and PEEP ≤8 cm H2O, the helmet can be discontinued and patient switched to high-flow nasal cannula, or oxygen supply at O2 at 6L/min or higher.

Helmet NIV could be resumed at any time if the respiratory rate was greater than 25 breaths/min and/or SpO2 was lower than 92% on FiO2 of ≥60%.

**Nursing care**

- Perform oral care/suction before helmet application.
- Place a nasogastric tube before helmet application if felt necessary by treating physician.
- A suction Yankauer for suctioning and a straw for drinking can be introduced through the patient port without removing the helmet.
- Intravenous sedation (Dexmedetomidine or others) can be used at the discretion of the treating physician to assure patient comfort.

**Equipment required**

- Mechanical ventilator (BP 840, PB 980, Servo-I, Drager V 500, Astral and V680)
- Unheated Double lumen circuit
- Bacterial/viral filter
- Subsalve helmet or equivalent
- Under arm pads (any kind)
- Ear plugs

**General recommendations to consider intubation for patients on NIV (assessed within 4 hours and at frequent intervals throughout NIV treatment):**

- Neurologic deterioration (not attributed to sedation)
- Persistent or worsening respiratory failure of NIV:
  - oxygen saturation <88%
  - respiratory rate >36/min
  - P/F ratio <100
  - a persistent requirement of FiO2 ≥70%
- Intolerance of face mask or helmet
- Airway bleeding
- Copious respiratory secretions
- Respiratory acidosis with pH <7.25
- Hemodynamic instability
- Significant radiologic worsening

**Humidification**

Appropriate level of humidification can be achieved via bubble humidifier with external oxygen flow of 5 L/Min entrained into the ventilator circuit proximal to the patient helmet.

**Case report form instructions**

**INTRODUCTION**

This document provides instructions for the Helmet study website, electronic Case Report Form (eCRF) and data definitions. It is designed to accompany the case report form (CRF) and the electronic case report form (eCRF).

The paper CRF can be used as a tool to collect data from the source documents and then entered into the web CRF. Working documents should be kept.

Bookmark this site on your browser for easy access and save as a shortcut on the desktop. Each time you log into the system, you must enter your username and password.

**Enrolling A Patient (Patient Initial Data and Screening Criteria)**

- Patients who meet all inclusion criteria and no exclusion criteria should be randomized and followed for up to 28 days in ICU OR until death OR withdrawal from the study. To randomize a patient, the data below will need to be entered into the REDCap by the site staff.
  - Patient No. (Starting with 01, 02, and so on)
  - Patient Initials
  - Enrollment Date
  - Date of Birth (If exact date of birth is not known initially, but age is considered to be greater than 14 or the ICU cut off age at your site, enter DOB as 01/01/year of birth or estimated year of birth)
  - Gender
- **In the Consent Section, select “No” or “Deferred” to continue with randomization.**
- If “Yes” was selected, this form will automatically lead to Eligible Non Randomized Form. **DO NOT FILL this form for patients who are randomized.**
- Please ensure to select “Complete” in Form Status and Save the form.
- If a patient is inadvertently randomized twice, please notify the Coordinating Center.
- If the patient was ineligible, but randomized, immediately inform the Coordinating Center and document a note to file.

**General points for data collection:**

**Baseline**

- Baseline data should be collected from the time PRIOR TO or IMMEDIATELY PRIOR TO THE TIME OF RANDOMISATION.
- DO NOT record data that was obtained AFTER the patient was randomized, as this may affect study results.

**Day 1**

- Study day 1 is from the time of randomization and 24 hours after i.e. if a patient is randomized on 01/01/2021 at 14:00H
- Study Day 1: time of randomization 01/01/2021 (14:00H) until 02/01/2021 (14:00H)
- Study Day 2: 02/01/2021 (14:00H) until 03/01/2021 (14:00H) And so on…
- If the patient is discharged from ICU, complete data for that day (from the start of your ICU chart day) up to the time of ICU discharge.

**Form Status**

Once form is completed, select “Complete” and save the form.

**BASELINE, DEMOGRAPHICS & COMORBIDITIES & RISK FACTORS**

1. Enter date of hospital admission
2. Enter date of ICU admission
3. Height: Record height in centimeters
4. Weight: Record weight (in kgs) on ICU admission.
5. Enter the Acute Physiology and Chronic Health Evaluation II score (APACHE II) calculated with the worst physiological scores in the **f*irst 24 hours of ICU admission***. See appendix for APACHE II Worksheet, or click the link for online APACHE II score calculation. *For manual calculation, go to page 8 of this manual.*
6. Check the appropriate responses for ***Comorbidities and Risk Factors***.

**RESPIRATORY SUPPORT AT BASELINE (24 HOURS BEFORE RANDOMIZATION)**

1. Select the appropriate oxygenation mode the patient was on right prior to randomization
2. High-flow oxygen delivered via nasal prongs or cannula by a specialized device, with FiO2 ≥0.4 at a flow rate of at least 30 L/min
3. For CPAP or BIPAP, please document the type including pressure support level and PEEP.
4. Select the FiO2 (in %)at the time of blood gas, if not applicable, please select the highest FiO2 for the day

**Functional assessment at baseline**

1. Fill this form at the time of randomization. This data point can be collected when consenting the patient.

**Dyspnea scale at baseline**

1. Complete/collect this at the time of randomization.

**DAILY DATA OF NON-INVASIVE RESPIRATORY SUPPORT(CONTINUE UNTIL THE DAY OF INTUBATION**

1. Timing of the first 24 hours start at the time of randomization for eg. Patient was randomized on 2/3/2021 @ 13:15

- First 24 hours 2/3/21 @13:15 to 3/3/21 @13:15
- Second 24 hours 3/3/21 @13:16 to 4/3/21 @13:15

1. Oxygen conversion mode


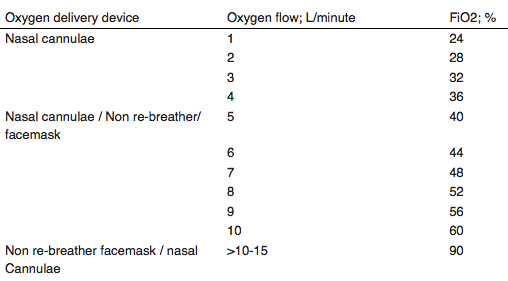


1. **Make sure that all oxygen support modes add up to 24 hours. Duration in minutes can be expressed in decimals e.g 12 hours and 30 minutes is entered as 12.5**
2. For respiratory rate, Sp02, and Fio2 take 4 hrs after enrollment and take on the same time for days 2, 3 and 4.
3. Dyspnea rating


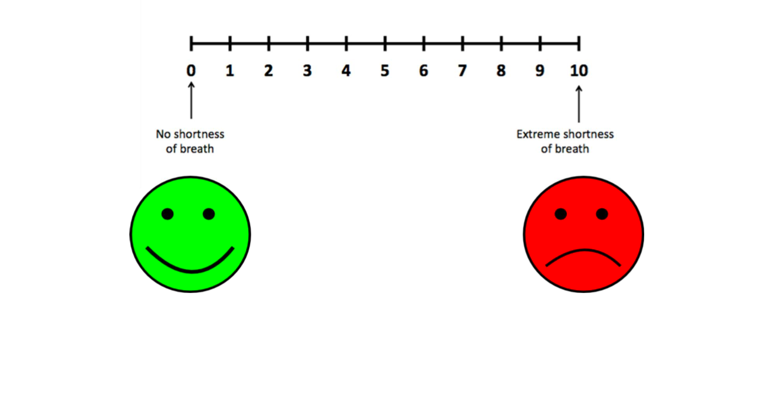


1. Device discomfort rating


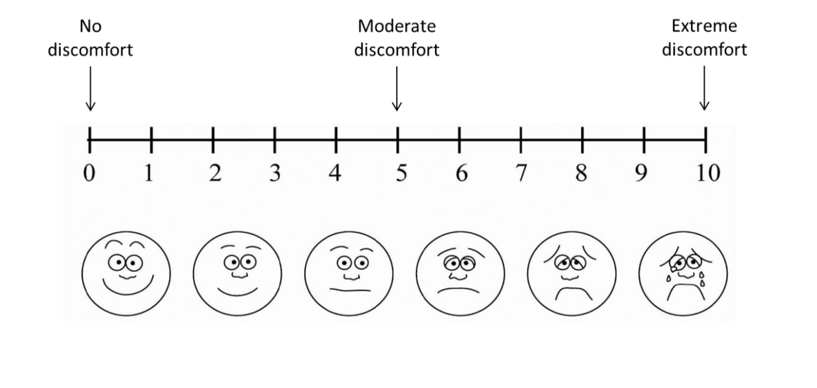


**USE OF HELMET (HELMET ARM)**

1. Total hours of helmet use – calculate total hours of helmet use until patient is intubated/ discharged. Duration in minutes can be expressed in decimals e.g 12 hours and 30 minutes is entered as 12.5.

**DAILY DATA UP TO 28 DAYS IN ICU**

1. Fill while patient is admitted to ICU, if patient moves to the ward or is discharged stop collecting this form.
2. If patient is mechanically ventilated, please continue to fill this form including data collection for barotrauma (pneumothorax, mediastinal air, subcutaneous emphysema or others).

**INVASIVE VENTILATION DATA**

1. Take the highest value for all variables during the first 24 hours of intubation
2. For Tidal Volume take the Machine Tidal Volume

**SEQUENTIAL ORGAN FAILURE ASSESSMENT (SOFA)**

- Fill in the columns of PaO_2_/FiO_2_ Ratio, Hypotension Score and Platelet Count for the corresponding **Study Days** (Day 1, 2, 3, 5, 7, 14, 21, 28).
- PaO_2_ stands for the partial pressure of O_2_ in arterial blood, measured in mmHg while FiO_2_ is the fraction or percentage of oxygen in the space being measured. To calculate for the ratio, divide the PaO_2_ value by the
- To calculate for the PaO_2_/FiO_2_ Ratio, divide the PaO_2_ by the FiO_2_. See appendix for conversion tables on estimating PaO_2_ and FiO_2_.

**ANTIMICROBIAL & COVID 19 MEDICATIONS (DURING ICU STAY UP TO 28 DAYS)**

**OUTCOMES**

1. To be filled on day 28 (from randomization)
2. Covid-19 results > first result (even if before hospital admission)
3. If patient is on continuous Dialysis document the study enrollment date

**180 DAY OUTCOME**

- Collected after 180 days from randomization via a phone call. Please fill the 180- follow up cover page and file in the master file as a source document.

**Co-interventions**

- Please select/tick the appropriate boxes corresponding to the interventions/treatments that the patient received from this ICU admission until day 28

**Protocol Violation**

- Complete this form only when applicable
- Document the deviation/violation that were seen as such.
- Also include the reason for the deviation/violation and the consequences (if any)

**Final Status**

- Document the final status of the patient by filling out the fields and selecting the appropriate responses
- Please do not forget to include data on co-enrolment

**Adverse Event Directly Related to the Study**

- Complete this form only when applicable
- Please report to the Coordinating Center immediately after the SAE occurs as per the guidelines of ICH-GCP.
- Indicate the date and time as to when the serious adverse event occurred
- Fill out the investigations done, treatment carried out and ask for a brief description of the serious adverse effect from the Intensivist
- Also ask for the signatures of the Intensivist and the Site Investigator with corresponding dates in this form and upload in the REDCap system.

**Withdrawal from the Trial**

- Select if the patient withdraws from the trial or not
- If yes, document the date and reason for the withdrawal from the trial

**APPENDIX I – APACHE SEVERITY OF DISEASE CLASSIFICATION**

**APACHE II CALCULATION SHEET**

The APACHE II scores is derived from 3 scoring systems: **Part A –** Acute Physiology Score, **Part B –** Age Points**, Part C –** Chronic Health Points

**Part A – Acute Physiology Score**

For each of the 12 physiological variables, select the most deranged value in the 24 hours prior to randomization. Enter the value in the right hand column.

For exact non-integer data that is not found in any of the given ranges, round the figure up or down to the nearest whole number. Eg, 44 years and 3 months is rounded down to 44 years and assigned 0 points; a calculated MAP of 129.7 is rounded up to 130 and assigned 3 points. For integers of xx**.**5 always round upwards. This is an arbitrary decision but must be followed for every patient to ensure consistency.

1. **Temperature** – this should be a core temperature measurement (rectal, tympanic, esophageal or via PAC). Where this is not possible, add 0.50C to the oral or axillary temperature
2. If mean arterial pressure (**MAP**) is not calculated by monitoring equipment, use the manual sphygmomanometer recording of systolic (SBP) and diastolic blood pressure (DBP) to obtain MAP using this equation MAP = (DBP x 2) + SBP ÷ 3.
3. If the patient has an atrial arrhythmia, measure the ventricular response rate (R waves) only to record the heart rate.
4. *A* – *aD*O2 is the difference between the calculated alveolar oxygen tension and the arterial oxygen tension. The alveolar oxygen tension is calculated by this equation: *A*O2 = 713 x *Fi*O2 – PaCO2 x 1.25. The *Fi*O2 here is expressed as a proportion of a unit. e.g. 100% *Fi*O2 = 1 and 60% equals 0.6. If the *FI*O2 (inhaled oxygen concentration) is greater than 50%, record the most deranged value for the *A* – *aD*O2. If the *FI*O2 is less than 50% record only the *Pa*O2 (arterial oxygen pressure). All measurements are in mmHg.
5. If ABGs have not been performed, choose the most deranged value for the serum venous bicarbonate (HCO3) in place of the **arterial pH**
6. If abnormal **serum creatinine** values reflect ARF as opposed to chronic renal failure then the points assigned to the creatinine values should be doubled. ARF is defined as any creatinine value that is not within the normal range designated by the APACHEII system. Thus for the purposes of this study, if the patient has any points for an increased creatinine any they are not documented to have chronic renal failure, then the creatinine points should be doubled.
7. To obtain a score for the Glasgow Coma Scale (**GCS**) use the GCS worksheet provided and subtract the GCS score from 15 to arrive at a score on the APACHE worksheet. Whenever possible, make an attempt to obtain a score for each physiological variable. If one of the 12 variables is not available, assign 0 (*zero*) points and make a note of this absence on the APACHE II worksheet. The assumption being made is that a test or measurement was not ordered because the status of the patient did not warrant investigation, rather than the data was missing.

To complete **Part B** – assign points to the age range that the patient fits in to. eg, a 48 year old patient

would be assigned 2 points.

To complete **Part C** – first decide if the patient meets any of the criteria provided on the worksheet for a history of severe organ insufficiency or immunocompromised. If there is no history, assign 0 points. If there is a history, assign points depending on whether the patient is an non-operative emergency admission or an emergency post-operative admission

Finally, add the points recorded for each of the 3 parts and enter the total score. The minimum score is 0 and the maximum score is 71. Keep the completed APACHE II worksheet in the documentation folder for this patient. It may be used for quality assurance measures. You will therefore need to print your hospital ID, Patient Initials and Patient Study Number on the APACHE worksheet.
